# Breast cancer mortality among women who have attended BreastScreen Norway, 1996–2023

**DOI:** 10.1186/s13058-026-02244-5

**Published:** 2026-03-17

**Authors:** Nataliia Moshina, Jonas Gjesvik, Xavier Castells, Marthe Larsen, Giske Ursin, Solveig Hofvind

**Affiliations:** 1 Department of Breast Screening, The Cancer Registry of Norway, Norwegian Institute of Public Health, Oslo, Norway; 2 Department of Medical Epidemiology and Biostatistics, Karolinska Institute, Solna, Sweden; 3 Department of Epidemiology and Evaluation, Hospital del Mar Research Institute, Barcelona, Spain; 4 University of Pompeu Fabra, Barcelona, Spain; 5 Network for Research on Chronicity, Primary Care, and Health Promotion (RICAPPS), Barcelona, Spain; 6 The Cancer Registry of Norway, Norwegian Institute of Public Health, Oslo, Norway; 7 Department of Nutrition, Institute of Basic Medical Sciences, University of Oslo, Oslo, Norway; 8 Department of Preventive Medicine, University of Southern California, Los Angeles, CA USA; 9 Department of Health and Care Sciences, Faculty of Health Sciences, UiT The Arctic University of Norway, Tromsø, Norway

**Keywords:** Mortality, Breast cancer, Screening, Self-selection bias, Follow-up

## Abstract

**Background:**

Organized mammographic screening has been shown to reduce breast cancer mortality across numerous high-quality studies. However, advancements in treatment, changes in screening techniques and adherence might alter the mortality reduction. In this study, we used data from an organized breast cancer screening program that started 30 years ago to investigate breast cancer mortality among women who were invited and attended the program (screened) versus women who were invited, but who did not attend (unscreened).

**Methods:**

This retrospective cohort study included information on invitation to and attendance in BreastScreen Norway, breast cancer diagnosis, emigration, death, and cause of death for women residing in Norway between January 1, 1996, and December 31, 2023. A total of 1,192,148 women aged 50–69 years without diagnosis of breast cancer at first invitation to screening and inclusion in the study were followed until breast cancer death, death of other causes, emigration or end of follow-up. We applied a Poisson regression model to compare incidence-based breast cancer mortality rate ratios (MRR) with 95% confidence intervals (CI) between screened and unscreened women invited to attend the program, adjusting for age, calendar year, time since cohort entry, and self-selection bias using correction coefficients based on Norwegian data, and a coefficient proposed by Duffy in 2002.

**Results:**

The screened cohort comprised 14,223,189 women-years, while the unscreened cohort accounted for 2,474,340 women-years. The crude breast cancer mortality rate was 28.2 per 100,000 women-years in the screened cohort, compared to 51.2 per 100,000 women-years in the unscreened cohort. The resulting unadjusted mortality rate ratio (MRR) was 0.55 (95% CI: 0.52–0.59). After adjusting for self-selection bias, the MRR remained strongly in favor of attending screening, and varied from 0.49 (95%CI 0.46–0.53) to 0.71 (95% CI: 0.66–0.76) when using self-selection bias correction coefficients derived from Norwegian data and was 0.61 (95% CI: 0.56–0.65) when using the coefficient provided by Duffy.

**Conclusion:**

After 27 years of follow-up, breast cancer mortality was 30–50% lower for invited and screened women compared to invited but unscreened women in BreastScreen Norway.

**Supplementary Information:**

The online version contains supplementary material available at 10.1186/s13058-026-02244-5.

## Introduction

Breast cancer is the most common cancer type in women worldwide, affecting over two million women each year and resulting in prominent mortality rates [1]. Studies have shown that breast cancer mortality is reduced by about 20% due to implementation of organized mammographic screening [2, 3], while the mortality reduction is shown to be about 40% among those screened [3, 4]. However, the reduction in mortality due to screening is affected by improved treatment over time [3, 5, 6]. As treatment advances are continuous, the effect of screening might be expected to stabilize possibly at a lower level over time or even decline [7, 8]. Therefore, we consider updated breast cancer mortality estimates to be valuable both for evaluating the effectiveness of mammographic screening and for assessing potential changes in the impact of treatment. Furthermore, in accordance with the European guidelines on breast cancer screening and diagnosis, reducing breast cancer mortality is the primary objective of mammographic screening; therefore, this outcome should be measured regularly within organized screening programs [2, 9].

When estimating the impact of screening on breast cancer mortality reduction, a follow-up period of more than 20 years provides a broader view of the screening effect, identifying possible time-related changes and accounting for more women with a longer survival time than a shorter follow-up time [2, 10, 11]. Studies on breast cancer mortality reduction among attenders versus non-attenders in mammographic screening programs with more than 20 years of follow-up are sparse, but data from the UK suggest that the breast cancer mortality reduction remains at 38–39% in participants [12, 13]. In a previous study from BreastScreen Norway with a follow-up time of 15 years, we found a 43% breast cancer mortality reduction among screening attenders [14, 15]. Studies on breast cancer mortality reduction in association with participation in mammographic screening are prone to self-selection bias [16, 17]. This type of bias implies that women not attending screening have lower education and health-awareness, and more comorbidities or disabilities, resulting in more aggressive disease and increased risk of breast cancer mortality [18–20].

This study was conducted to evaluate the effect of an organized breast cancer screening program, BreastScreen Norway, on breast cancer mortality after 27 years of follow-up. More specifically, we aimed to explore the effect of screening attendance versus non-attendance (screened versus unscreened) on breast cancer mortality, while adjusting for self-selection bias.

## Materials and methods

### Study setting

BreastScreen Norway is an organized screening program inviting all women aged 50–69 residing in Norway to biennial mammographic screening [21]. The program started in 1996 and became nationwide in 2005 [21]. The attendance rate has remained about 75% since the start of the program [21]. In Norway, cancer reporting has been mandatory by law since 1952 [22]. The Cancer Registry receives information on all cancers from clinicians, pathologists, hospitals and institutions, as well as from death certificates, with 0.2% of all cancers ascertained only from death certificates [4]. The unique 11-digit personal identification number of each citizen in Norway facilitates the follow-up for cause specific mortality. For this study, we obtained individual data on dates of screening invitation, participation, and breast cancer diagnosis from the Cancer Registry of Norway, Norwegian Institute of Public Health, and breast cancer death and cause of death from the Norwegian Cause of Death Registry. Breast cancer mortality has been reported as part of quality control and has been previously estimated in studies from Norway [3, 4, 23].

### Study population

**Fig. 1:**
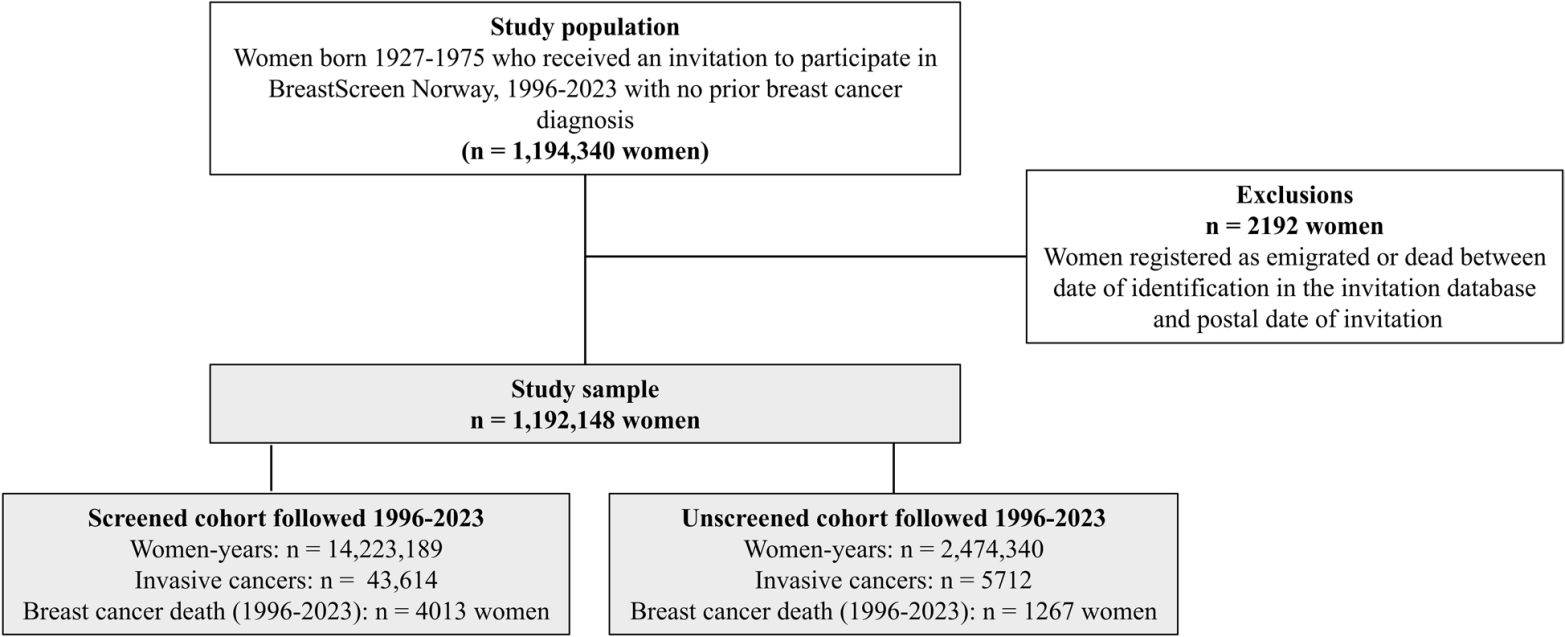
Flow-chart of the study population. Note: For bilateral breast cancer, the first breast cancer diagnosed during the study period was included

We included data from all women invited to the program during the period from January 1, 1996, to December 31st, 2023. Only women without breast cancer prior to receiving the first invitation to BreastScreen Norway were included in the study population. Women who emigrated, died or were diagnosed with breast cancer between date of identification in the invitation database and postal day of invitation were excluded (n = 2192) (Fig. 1). This left data from n = 1,192,148 women available for analyses. Further, the study population was divided into two cohorts, the screened and unscreened cohort. The screened cohort consisted of women who attended BreastScreen Norway at least once, while the unscreened cohort received one or more invitations, but did not attend screening during the study period. Women were included in the unscreened cohort from postal date of their first invitation to BreastScreen Norway, and women were allocated to the screened cohort from the date of their first screening. The time each woman contributed to each of the two cohorts was presented in women-years. Allocation to the screened cohort excluded the possibility of allocation to the unscreened cohort at a later time-point. Women who initially were in the unscreened cohort and then later chose to attend, contributed with women-years to both cohorts. This resulted in shorter follow-up for the unscreened versus screened cohort. Women were followed until emigration resulting in loss of follow-up, death of any cause, or the end of follow-up, December 31, 2023.

This retrospective cohort study followed the Strengthening the reporting of observational studies in epidemiology (STROBE) cohort reporting guidelines [24] (Online Appendix B).

### Variables of interest

Breast cancer was defined as invasive breast cancer. Women diagnosed with ductal carcinoma in situ (DCIS) were not included in the analyses.

Women registered with invasive breast cancer as primary cause of death contributed to breast cancer mortality. Other causes of death were registered as end of follow-up. Age at inclusion was defined as the age of the woman at the time of the first screening examination for the screened cohort and at the time of first invitation for the unscreened cohort. Attained age was calculated for each woman as the number of years from study inclusion to end of follow-up. Years since inclusion in the cohort were defined as the time (years) from inclusion in the group (screened and unscreened) to the end of follow-up.

### Estimating the self-selection bias coefficient using Norwegian data

Since screened women were expected to be healthier than unscreened women [20], adjustment for self-selection bias was performed using a correction coefficient described by Duffy et al. [16]. The mortality rate ratio between screened women and unscreened women who were considered “would be screened” (Ψ′) was calculated from the formula shown in Duffy et al. [16]

$$ \Psi^{\prime} = \Psi \left( {\frac{p*D}{{\left[ {1 - \left( {1 - p} \right)D} \right]}}} \right), $$

where, Ψ was the estimated relative risk of breast cancer death for screened compared to unscreened women, p was the proportion of women screened after any invitation, and D, the self-selection bias correction coefficient, was the relative risk of breast cancer death for unscreened women compared to an uninvited comparison group. In our study, the estimated relative risk of breast cancer for screened women compared to unscreened women (Ψ) was 0.42. Further, the proportion of women screened after at least one invitation during the study period (p) was 86% (1,023,567/1,192,148). We calculated D based on Norwegian data. We used the mortality rate for the invited but not screened women in our study as the numerator (mortality rate for unscreened women, 51.2 per 100,000 women-years). To find the denominator (mortality rate in an uninvited comparison group), we used information for the uninvited groups aged 50–88 years from the follow-up model of the study by Sebuødegård et al., investigating breast cancer mortality associated with implementation of mammographic screening in Norway [3]. In the screening period, their cohort was divided into invited and non-invited women, as the staggered implementation of the screening program in Norway was conducted in 1996–2005, while in the prescreening period (1977–1995), the uninvited women were divided into pseudo-invited and pseudo-non-invited [3]. The mortality rate in the uninvited groups was therefore estimated to be 33.3 per 100,000 women-years in the screening period (1996–2014), 46.8 per 100,000 women-years for pseudo-non-invited women in the prescreening period (1977–1995) and 49.6 per 100,000 women-years for pseudo-invited women in the prescreening period (1977–1995) (Table A2).

### Statistical analysis

We used descriptive statistics to describe the study population. Numbers of women, follow-up time, breast cancer diagnosis (for invasive breast cancer), breast cancer mortality rate (MR) and breast cancer mortality rate ratio (MRR) according to age at inclusion in the cohorts (< 55, 55–59, 60–64, 65+ years) were provided in Online Appendix A (Table A1). MR was estimated as the number of breast cancer deaths divided by the number of women-years. Poisson regression was used to estimate the MRR for screened versus unscreened cohort, mutually adjusted for attained age (years), calendar year, and years since inclusion in the cohort. The self-selection bias correction coefficient was 1.36 using the estimates proposed by Duffy [16]. The coefficient was 1.54 based on the most recent Norwegian data from non-invited women in the screening period (1996–2014), and 1.14 when including the data from non-invited women in the prescreening (1977–1995) and screening period (1996–2014) (Table A2). Crude and cumulative MR were presented. Stata version 18 was used for conducting statistical analyses (StataCorp. 2023. Stata 18. Statistical Software. College Station, TX: StataCorp LLC).

## Results

The study population included data from 1,192,148 women contributing to 16,697,529 women-years, 14,233,189 women-years in the screened cohort and 2,474,340 in the unscreened cohort (Fig. 1). A total of 43,614 invasive breast cancers were diagnosed, and 4013 breast cancer deaths occurred in the screened cohort, while it was 5712 invasive breast cancers diagnosed and 1267 breast cancer deaths in the unscreened cohort.

Crude MR was 28.2 (95% CI: 27.4- 29.1) for the screened cohort and 51.2 (95% CI: 48.5–54.1) for the unscreened cohort, resulting in an MRR of 0.55 (95% CI: 0.52–0.59) (Table 1). When adjusting for attained age, calendar year, and number of previous follow-up years in cohort, the screened cohort had a mortality reduction of 58% compared to the unscreened cohort (MRR of 0.42, 95% CI: 0.39–0.45) (Tables 1, 2). The MRR decreased by 3% per calendar year (MRR of 0.97, 95%CI: 0.96–0.97) but increased by 5% with each attained year of age (MRR of 1.05, 95%CI: 1.04–1.05) in the model (Table 2). Further, MRR increased by follow-up time since diagnosis.

**Table 1:** Number of breast cancers (BC) and BC deaths, follow-up in women-years (wy), crude BC mortality rate (MR), and crude and adjusted BC mortality rate ratio (MRR) with 95% confidence intervals (CI) for the screened and unscreened cohort of women invited to BreastScreen Norway, 1996–2023

Cohorts	BC cases (n)	BC deaths (n)	Follow-up time (wy)	BC MR/ 100,000 wy (95% CI)	BC MRR/ 100,000 wy (95% CI)	Adjusted ^€^ BC MRR/ 100,000 wy (95% CI)	Adjusted^€ *^ BC MRR/ 100,000 wy (95% CI)	Adjusted^€ #^ BC MRR/ 100,000 wy (95% CI)	Adjusted^€ β^ BC MRR/ 100,000 wy (95% CI)
Unscreened	5712	1267	2,474,340	51.2 (48.5–54.1)	1.00	1.00	–	–	–
Screened	43,614	4013	14,223,189	28.2 (27.4–29.1)	0.55 (0.52–0.59)	0.42 (0.39–0.45)	0.61 (0.47–0.78)	0.71 (0.64–0.78)	0.49 (0.46–0.53)

^€^Adjusted for attained age, calendar year, and number of previous follow-up years in cohort divided into 3-year intervals

^*^Adjusted for self-selection bias of attending women using the method described in Duffy et al.[16]

^#^Adjusted for self-selection bias of attending women using Norwegian data for non-invited women during the period 1995–2014

^β^Adjusted for self-selection bias of attending women using Norwegian data for non-invited women during the period 1977–2014

**Table 2:** Breast cancer (BC) the cohort (3-year groups), and attained age, 1996–2023mortality rate ratio (MRR) per 100,000 women years (wy) with 95% confidence intervals (CI) for screened compared to unscreened cohort of women invited to BreastScreen Norway adjusted for number of years since inclusion in the cohort, previous follow-up time in

	BC MRR per 100,000 wy (95% CI)^¤^		*p*-value
Unscreened cohort	1	–	–
Screened cohort	0.42	(0.39–0.45)	< 0.001
Calendar years since 1996	0.97	(0.96–0.97)	< 0.001
Follow-up time in cohort (years)			
0–2	1	–	–
3–5	2.88	(2.48–3.33)	< 0.001
6–8	3.97	(3.42–4.61)	< 0.001
9–11	4.40	(3.76–5.16)	< 0.001
12–14	5.23	(4.42–6.20)	< 0.001
15–17	5.88	(4.89–7.08)	< 0.001
18–20	6.31	(5.15–7.73)	< 0.001
21–23	7.70	(6.17–9.62)	< 0.001
24–26	9.72	(7.56–12.50)	< 0.001
27–29	10.53	(2.59–42.89)	0.001
Attained age (years)	1.05	(1.04–1.05)	< 0.001

^¤^mutually adjusted (for attained age (years), calendar year, and years since inclusion in the cohort)

Applying the self-selection bias correction coefficient estimated by Duffy [16] resulted in a 6 percentage points lower mortality reduction, suggesting 39% lower breast cancer mortality (MRR: 0.61, 95%CI: 0.47–0.78) for the screened compared to unscreened cohort (Tables 1, Table A1). The reduction in breast cancer mortality among the screened women varied from 29% (MRR: 0.71, 95% CI: 0.66–0.76) to 51% (MRR: 0.49, 95% CI: 0.46–0.53) when using self-selection bias correction coefficients estimated Norwegian data for the period of 1995–2014 and 1977–2014, respectively (Tables 1, A2).

**Fig. 2:**
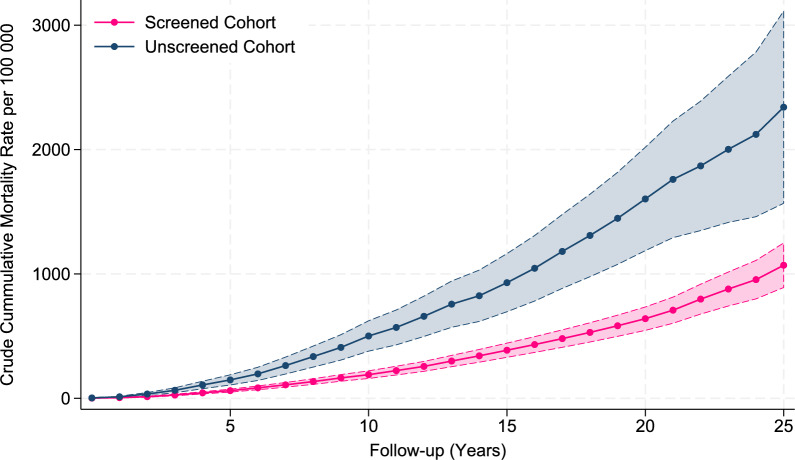
Crude cumulative breast cancer mortality rates with 95% confidence intervals per 100,000 women-years for the screened and unscreened cohorts of women invited to BreastScreen Norway by time since inclusion in the cohorts, 1996–2023

The crude and cumulative breast cancer MR increased constantly in both cohorts and was lower in the screened cohort compared to the unscreened cohort from year 1 until the end of follow-up (Table 3, Fig. 2).

**Table 3:** Follow-up time (women-years, wy), number of breast cancer (BC) deaths, BC mortality rate (MR) per 100,000 wy with 95% confidence interval (CI), and age by previous follow-up time for the screened and unscreened cohort of women invited to BreastScreen Norway, 1996–2023

Previous follow-up (years)	Screened cohort				Unscreened cohort							
	Follow-up time (wy)	BC deaths (n)	Crude BC MR (per 100,000 wy, 95%CI)		Cumulative BC MR (per 100,000 wy, 95%CI)		Follow-up time (wy)	BC deaths (n)	Crude BC MR (per 100,000 wy, 95%CI)		Cumulative BC MR (per 100,000 wy, 95%CI)	
0	1,007,491	17	1.7	(1.1–2.7)	1.7	(0.9–2.5)	392,026	14	3.6	(2.1–6.0)	3.6	(1.6–5.5)
1	963,215	36	3.7	(2.7–5.2)	5.4	(3.5–7.4)	284,731	26	9.1	(6.2–13.4)	12.7	(7.2–18.2)
2	921,301	73	7.9	(6.3–10.0)	13.3	(9.6–17.1)	200,357	44	22.0	(16.3–29.5)	34.7	(22.7–46.6)
3	884,404	119	13.5	(11.2–16.1)	26.8	(20.7–32.9)	179,185	54	30.1	(23.1–39.4)	64.8	(44.8–84.8)
4	854,853	149	17.4	(14.8–20.5)	44.2	(35.4–53.1)	152,086	65	42.7	(33.5–54.5)	107.5	(77.2–137.9)
5	817,116	145	17.7	(15.1–20.9)	62.0	(50.2–73.7)	139,738	57	40.8	(31.5–52.9)	148.3	(107.4–189.3)
6	781,495	176	22.5	(19.4–26.1)	84.5	(69.4–99.6)	123,330	60	48.7	(37.8–62.7)	197.0	(143.7–250.3)
7	743,508	180	24.2	(20.9–28.0)	108.7	(90.1–127.3)	113,997	76	66.7	(53.3–83.5)	263.6	(195.2–332.1)
8	706,633	186	26.3	(22.8–30.4)	135.0	(112.7–157.4)	102,418	74	72.3	(57.5–90.7)	335.9	(251.0–420.8)
9	669,895	197	29.4	(25.6–33.8)	164.4	(138.0–190.9)	94,803	70	73.8	(58.4–93.3)	409.7	(307.6–511.9)
10	637,307	165	25.9	(22.2–30.2)	190.3	(159.9–220.7)	86,219	79	91.6	(73.5–114.2)	501.4	(379.1–623.7)
11	605,290	198	32.7	(28.5–37.6)	223.0	(188.2–257.9)	79,840	55	68.9	(52.9–89.7)	570.3	(429.7–710.8)
12	574,850	195	33.9	(29.5–39.0)	257.0	(217.4–296.6)	72,803	65	89.3	(70.0–113.9)	659.5	(497.2–821.8)
13	540,922	232	42.9	(37.7–48.8)	299.9	(254.8–344.9)	66,635	65	97.5	(76.5–124.4)	757.1	(571.1–943.1)
14	508,785	214	42.1	(36.8–48.1)	341.9	(291.1–392.7)	60,473	41	67.8	(49.9–92.1)	824.9	(618.1–1031.7)
15	475,913	214	45.0	(39.3–51.4)	386.9	(330.0–443.7)	55,210	58	105.1	(81.2–135.9)	929.9	(696.1–1163.8)
16	443,479	203	45.8	(39.9–52.5)	432.7	(369.5–495.8)	50,093	58	115.8	(89.5–149.8)	1045.7	(782.1–1309.3)
17	411,094	196	47.7	(41.5–54.8)	480.3	(410.6–550.1)	45,732	62	135.6	(105.7–173.9)	1181.3	(884.0–1478.6)
18	374,252	187	50.0	(43.3–57.7)	530.3	(453.3–607.3)	41,281	53	128.4	(98.1–168.1)	1309.7	(977.9–1641.5)
19	327,576	175	53.4	(46.1–62.0)	583.7	(498.9–668.6)	35,508	49	138.0	(104.3–182.6)	1447.7	(1077.2–1818.1)
20	276,375	157	56.8	(48.6–66.4)	640.5	(546.8–734.2)	28,912	45	155.6	(116.2–208.5)	1603.3	(1187.4–2019.2)
21	211,050	144	68.2	(58.0–80.3)	708.8	(603.9–813.6)	21,607	34	157.4	(112.4–220.2)	1760.7	(1291.9–2229.5)
22	154,038	137	88.9	(75.2–105.2)	797.7	(677.9–917.5)	15,643	17	108.7	(67.6–174.8)	1869.4	(1348.8–2389.9)
23	114,318	93	81.4	(66.4–99.7)	879.0	(742.8–1015.3)	11,286	15	132.9	(80.1–220.5)	2002.3	(1414.5–2590.1)
24	88,873	67	75.4	(59.3–95.8)	954.4	(800.2–1108.7)	8,312	10	120.3	(64.7–223.6)	2122.6	(1460.3–2784.9)
25+	129,155	158	122.3	(104.7–143.0)	1076.8	(898.0–1255.5)	12,117	15	173.3	(113.0–265.8)	2295.9	(1523.0–3068.7)
Total	14,223,189	4013	28.2				2,474,340	1267	51.2

## Discussion

In this study, including 27 years of follow-up after breast cancer diagnosis, breast cancer mortality was lower for women invited and screened in BreastScreen Norway compared with those invited but not screened, when adjusting for self-selection bias. The reduction varied from 29 to 54% depending on which estimate was used as the self-selection bias correction coefficient. Our results are in line with previous studies from Norway and other organized screening programs [2, 10, 12, 14, 25]. Compared to previous studies with a shorter follow-up, our study demonstrates that the mortality reduction associated with screening attendance is still marked and present after more than 20 years [2, 10, 12, 14, 25]. The findings indicate that participation in BreastScreen Norway is beneficial for women in terms of reduced risk of breast cancer death over time.

Studies have shown that women attending breast cancer screening are healthier, have more favorable sociodemographic status and lifestyle than women who do not attend [20, 26]. Adjusting for self-selection bias is thus crucial in mortality analyses [10, 13, 16, 27]. However, this adjustment is also challenging because it involves a number of assumptions [16]. A method provided by Duffy et al. [16] includes parameters that could be measured locally. An important parameter is breast cancer mortality among non-invited women in the same age as the target group of the screening program. In a nationwide program where all women are invited, this means that data from the period before the screening program started should be used [16], suggesting that improvements in breast cancer treatment during the last 30 years might not be accounted for [16, 28]. Gradual implementation of BreastScreen Norway during the period from 1995 to 2005 facilitated the use of information on uninvited women from that period [3]. These data, more than 20 years old, resulted in a self-selection bias coefficient of 1.54 and a mortality reduction of 29% for screened versus unscreened women. Most of the women (approximately 86%) who were invited but not screened 1995–2005, were screened later. Therefore, most of the women-years in the unscreened group was among young women. Further, the age distribution among the unscreened was heavily skewed towards the younger side [3]. This lowered the mortality rate among the unscreened group and thus resulted in a conservative estimate of the self-selection bias coefficient. To address this issue, the summary self-selection bias estimate of 1.14 (95%CI: 1.07–1.21) was introduced based on Norwegian data from the period 1977–2014 [3]. We assume this coefficient to be representative for the Norwegian unscreened and non-invited women as it falls within the 95% CI of 1.11–1.67 provided by Duffy et al. [16]. Our estimates of self-selection bias differed from an estimate of a recent quantitative evaluation of 1.78, suggesting overall overestimation of the screening effect in cohort studies adjusting for self-selection bias [17]. Despite lower values in our analyses, we consider a summary self-selection bias estimate of 1.14 relevant and sufficient for this study as individual data from Norway were used in the calculations.

Studies have analyzed the effect of age on breast cancer mortality reduction and reported either insignificant increase [29] or even decrease in breast cancer mortality with age, considering deaths from other causes and more indolent disease among older women as the main reasons for the findings [29, 30]. Our study showed an increase in breast cancer mortality with age which might be associated with lack of adjustment for competing causes of death but also prevailing of breast cancer as cause of death in older women in Norway [31]. Statistics from NORDCAN, a web-based tool providing data on cancer incidence, mortality, prevalence and survival in Nordic countries, indicate that women were about 10–15 years older at breast cancer death in 2023 compared to 1996 [32].

Breast cancer treatment has improved during the last decades, including less radical surgery and more effective adjuvant systemic treatment and standardized management approach [28, 33]. Although the treatment effect was not directly estimated in our study, we assume this to be a part of the 3% decrease in breast cancer mortality per calendar year. A previous study from Norway reported that a cumulative effect of improved treatment on breast cancer mortality reduction for women aged 50–69 years was 15–17% [3]. In addition, part of this effect might be related to a shorter follow-up in later years for all included women.

A Norwegian study comparing breast cancer mortality for screened and unscreened women with 15 years of follow-up showed MRRs similar to MRRs in our study, including crude (0.52), adjusted (0.39) and corrected with Duffy’s self-selection bias coefficient (0.57) [14]. These comparable results suggest that the impact of screening on breast cancer mortality reduction has not changed markedly regardless of the length of follow-up. However, when the absolute difference in risks and possible numbers of deaths avoided due to screening are considered, the impact of screening on breast cancer mortality reduction increases over time, as shown in Table 3.

We performed a cohort study. Although this is not the most optimal design, and an RCT would methodologically have been the most advantageous to assess the effect of screening on breast cancer mortality, running RCT’s with mammographic screening in 2025 will not be practical nor ethically acceptable. A systematic review of RCTs on breast cancer mortality reduction due to mammographic screening conducted 1963–2015 reported a 22% lower breast cancer mortality (with a relative risk of 0.78, 95%CI: 0.69–0.90) for invited versus non-invited women [11]. Our estimated breast cancer mortality reduction of 29–54% exceeds that reported in the review, likely reflecting differences in study design. Our per-protocol comparison of screened versus unscreened women contrasts with the intention-to-treat comparisons of invited versus non-invited women in randomized trials. A recent meta-analysis of cohort studies reported a pooled relative risk of 0.55 (95% CI: 0.50–0.60; I^2^ = 42%) for screened versus unscreened women, consistent with our findings [10]. However, the observed association between screening attendance and reduced all-cause mortality suggests that cohort designs may overestimate the effect of screening on breast cancer mortality [10].

Our study has limitations associated with lack of adjustment for possible confounders. Women who have opted out one or several rounds prior to their breast cancer diagnosis have been shown to encounter worse breast cancer outcomes compared to regular attenders [34], implying the necessity to adjust for screening attendance patterns in the estimation of breast cancer mortality. Socioeconomic position, immigrant status, parity, comorbidities, and all-cause and cause-specific mortality are important confounders in breast cancer mortality estimates [10, 27, 35–38] but could not be included due to data limitations. Low income, low educational attainment, and immigrant status have been associated with poorer breast cancer outcomes [35], and in Norway with lower attendance in BreastScreen Norway [26] and poorer cancer survival [36]. Lack of adjustment for these factors may therefore have led to overestimation of the observed breast cancer mortality reduction among screened women [39].

Similarly, lack of information on all-cause and other cause mortality may have resulted in overestimation of breast cancer mortality among non-attenders [10, 27, 39]. However, evidence is inconsistent: higher breast cancer mortality among women with higher socioeconomic status has been reported from European screening programs [40], and lower all-cause mortality among immigrants has been observed in Denmark [41], complicating interpretation of our findings.

High parity has a protective effect on breast cancer mortality in Norway [38] and failure to adjust for parity may have overestimated mortality among non-attenders, among whom immigrant background and low socioeconomic status, and thus high parity, were more prevalent [42, 43]. Finally, omission of comorbidities may have overestimated breast cancer mortality in both groups, as comorbid conditions can restrict treatment options [37].

Lead time, i.e. period between disease detection due to screening and the onset of symptoms, and overdiagnosis, i.e. detection of cancers that would not evolve to a clinical stage during the women life might result in excess mortality due to treatment effects [2, 44–48]. However, as the effect on excess mortality could not be measured directly [44], it was not considered in our analysis. We lacked data on potential misclassification of cause of death. Some deaths may have been incorrectly attributed to breast cancer if older women had a prior diagnosis of screen‑detected breast cancer, as previously demonstrated in a study from Sweden [49, 50]. Such misclassification could have led to an overestimation of breast cancer mortality among screened women. Further, confounders as competing causes of death and participation in opportunistic screening were not included and represent aspects which could influence the results in both directions, depending on the numbers [4, 51]. All the aforementioned factors are challenging in terms of inclusion in breast cancer mortality estimations as those require specific background information available during the long follow-up period. We assume, however, that our self-selection bias correction coefficients have partially taken this information into account. Strengths of the study are related to the long follow-up time in the nationwide program, individual data and high completeness of the data.

In conclusion, we found that breast cancer mortality was 30–50% lower for women invited and screened in BreastScreen Norway compared to women invited but never screened. The differences in breast cancer mortality for screened versus unscreened women continued to increase also after the first ten years with screening.

## Supplementary Information

Additional file1.

Additional file2.

## Data Availability

Research data used in the analyses is not publicly available. Data from the Norwegian mandatory national health registries can be requested from https://helsedata.no/, given the legal basis in Articles 6 and 9 of the GDPR and that the processing is in accordance with Article 5 of the GDPR, as well as Norwegian legislation.
