# Supplementary material for: Breast cancer mortality among women who have attended BreastScreen Norway, 1996–2023
Source: Breast Cancer Res. 2026 Mar 17;28:75. doi: 10.1186/s13058-026-02244-5 (PMC13107694; doi:10.1186/s13058-026-02244-5)
Supplement: Supplementary file 1 — Additional file1. [file 13058_2026_2244_MOESM1_ESM.docx]

Supplemental file

Table A1. Follow-up time, number of invasive breast cancers (BC) and BC deaths, BC mortality rates (MR) per 100,000 women-years (wy), number of women who changed from the unscreened to the to the screened cohort, and number of women who were alive at the end of follow-up or lost at follow-up for the screened and unscreened cohort of women invited to BreastScreen Norway by age at inclusion in the cohorts (<55, 55-59, 60-64, 65+ years),1996-2023

|  | Screened cohort | | | | | Unscreened cohort | | | | |
| --- | --- | --- | --- | --- | --- | --- | --- | --- | --- | --- |
|  | Age Groups (years) | | | | | Age Groups (years) | | | | |
|  | <55 | 55-59 | 60-64 | 65+ | Total | <55 | 55-59 | 60-64 | 65+ | Total |
| Women (n) | 726,776 | 132,164 | 91,127 | 73,500 | 1,023,567 | 890,256 | 124,565 | 94,643 | 82,684 | 1,192,148^β^ |
| Follow-Up Time (years) | 8,961,523 | 2,379,267 | 1,640,889 | 1,241,511 | 14,223,189 | 1,662,422 | 301,173 | 241,416 | 269,329 | 2,474,340 |
| BC (n) | 26,735 | 7839 | 5344 | 3696 | 43,614 | 3403 | 832 | 710 | 767 | 5712 |
| BC Deaths (n) | 1741 | 841 | 732 | 699 | 4013 | 601 | 219 | 212 | 235 | 1267 |
| MR (per 100,000 wy) | 19.4 | 35.3 | 44.6 | 56.3 | 28.2 | 36.2 | 72.7 | 87.8 | 87.3 | 51.2 |
| MRR^*^ | 0.54 | 0.49 | 0.51 | 0.65 | 0.55 |  |  |  |  |  |
| Year of Inclusion (mean) | 2011 | 2004 | 2003 | 2002 | 2008 | 2010 | 2001 | 2001 | 2001 | 2008 |
| Year of BC Diagnosis (mean) | 2015 | 2012 | 2011 | 2010 | 2013 | 2014 | 2011 | 2010 | 2010 | 2012 |
| Year of BC Death (mean) | 2015 | 2015 | 2015 | 2014 | 2015 | 2015 | 2013 | 2013 | 2012 | 2014 |
|  |  |  |  |  |  |  |  |  |  |  |
| Changed Group (n) | 0 | 0 | 0 | 0 | 0 | 772,554 | 107,969 | 79,544 | 63,500 | 1,023,567 |
| Alive at End of Follow-Up (n) | 684,923 | 104,826 | 58,305 | 30,282 | 878,336 | 98,624 | 8958 | 6532 | 5426 | 119,540 |
| Loss of Follow-Up^#^ (n) | 40,112 | 26,497 | 32,090 | 42,519 | 141,218 | 18,477 | 7419 | 8 355 | 13,523 | 47,774 |

*MR Ratio is Mortality Rate Ratio between screened and unscreened cohorts

^β^All women were included in an unscreened cohort because the period between invitation and screening attendance was computed as an unscreened cohort

^#^ Loss of follow-up was primarily associated with emigration

Table A2. Estimation of mortality rate ratio (MRR) associated with breast cancer screening attendance using various methods of adjustment for self-selection bias

|  | Our Cohort | Estimate from Swedish Trials [Duffy (2002)] | Prescreening Period (Pseudo-Non-Invited) [Sebuødegård (2020)] ^α^ | Screening Period (Non-Invited) [Sebuødegård (2020)] ^β^ | Prescreening Period (Pseudo-Invited) [Sebuødegård (2020)] ^γ^ | All Non-Invited Women [Sebuødegård (2020)] |
| --- | --- | --- | --- | --- | --- | --- |
| Mortality rate for unscreened women (per 100,000 women-years) | 51.2 | n.a. | n.a. | n.a. | n.a. | n.a. |
| Mortality rate for non-invited women (per 100,000 women-years) | n.a. | n.a. | 46.8 | 33.3 | 49.6 | 44.9 |
|  |  |  |  |  |  |  |
| D (estimated MRR between unscreened and non-invited) |  | 1.36  (1.11 – 1.67) | 1.09  (1.18 – 1.02) | 1.54  (1.66 – 1.42) | 1.03  (1.10 – 0.97) | 1.14  (1.21 – 1.07) |
| p (proportion of women screened after invitation) | 0.86 |  |  |  |  |  |
| Ψ (estimated MRR between unscreened and screened) ^δ^: | | | | | | |
|  | 0.42 |  |  |  |  |  |
| Ψ’ (estimated MRR between screened and would be screened) ^ε^: | | | | | | |
|  |  | 0.61  (0.47 – 0.78) | 0.47  (0.43 – 0.51) | 0.71  (0.64 – 0.78) | 0.44  (0.40 – 0.47) | 0.49  (0.46 – 0.53) |

n.a. - not applicable

α - Women in screening age assigned to the pseudo-non-invited group in the prescreening window, see Sebuødegård [2020](1) for details

β - Women in screening age who resided in areas were screening was not yet implemented in early years of the implementation of breast cancer screening in Norway

γ - Women in screening age assigned to the pseudo-invited group in the prescreening window, see Sebuødegård [2020](1) for details

δ - Adjusted for attained age, calendar year and number of previous follow-up years in cohort divided into 3-year intervals.

ε - Adjusted for self-selection bias using the method described by Duffy et al.(2))

References

1. Sebuødegård S, Botteri E, Hofvind S. Breast cancer mortality after implementation of organized population-based breast cancer screening in Norway. J Natl Cancer Inst. 2019;112(8):839-46.

2. Duffy SW, Cuzick J, Tabar L, Vitak B, Chen TH-H, Yen M-F, et al. Correcting for Non-Compliance Bias in Case–Control Studies to Evaluate Cancer Screening Programmes. Journal of the Royal Statistical Society Series C: Applied Statistics. 2002;51(2):235-43.
